# Dynamic changes in comorbid conditions following vagus nerve stimulation for epilepsy

**DOI:** 10.1186/s42494-025-00222-6

**Published:** 2025-05-30

**Authors:** Deng Chen, Lina Zhu, Ling Liu, Dong Zhou, Xintong Wu

**Affiliations:** 1 Department of Neurology, West China Hospital, Sichuan University, Chengdu, 610041 China; 2 Rehabilitation Medicine Center and Institute of Rehabilitation Medicine, West China Hospital, Sichuan University, Chengdu, 610041 China; 3 Key Laboratory of Rehabilitation Medicine in Sichuan Province, West China Hospital, Sichuan University, Chengdu, 610041 China

**Keywords:** VNS, Epilepsy, Comorbid Conditions

## Abstract

**Background:**

Vagus nerve stimulation (VNS) has been widely used in the clinical treatment of epilepsy, while its effects on comorbidities in epilepsy remain incompletely elucidated. This study aimed to evaluate the impact of VNS on comorbidities and quality of life in adult patients with epilepsy.

**Methods:**

A longitudinal, multicenter cohort study was conducted from 2021 to 2024 among adult patients with epilepsy who underwent VNS implantation. We enrolled 128 participants from 83 hospitals. The inclusion criteria were patients over 18 years old, diagnosed with epilepsy according to the 2014 International League Against Epilepsy guidelines, and having complete data from at least two follow-up visits. Standard assessment tools, including diagnosis according to International Classification of Diseases, 10th Edition (ICD-10), Neurological Disorders Depression Inventory for Epilepsy (NDDI-E), Generalized Anxiexy Disorde-7 (GAD-7), Pittsburgh Sleep Quality Index (PSQI), and Quality of Life in Epilepsy-31 (QOLIE-31) were used to evaluate comorbidities and quality of life. Statistical analysis was performed using SPSS 26.0. The major clinical measurements were changes in the scales above before and after VNS implantation during follow-up. Generalized estimation model was applied to illustrate the effect over time an its relation to seizure control.

**Results:**

A total of 113 participants met the inclusion criteria. Baseline characteristics were comparable between the comorbidity and non-comorbidity groups in terms of gender, seizure onset, age at VNS implantation, seizure types, or the number of antiseizure medications used. Significant improvements were observed from the implantation to the end of follow-up. The PSQI score decreased from 5.43 ± 3.60 to 4.44 ± 3.14 (*P* < 0.01), indicating better sleep quality. Depressive symptoms (NDDI-E) and anxiety symptoms (GAD-7) decreased significantly, with scores dropping from 6.49 ± 4.67 to 4.83 ± 4.37 (*P* < 0.01) and from 7.15 ± 5.06 to 4.95 ± 3.69 (*P* < 0.01), respectively. The QOLIE-31 score increased from 54.40 ± 15.70 to 61.33 ± 16.19 (*P* < 0.01), suggesting improved quality of life. Further analysis indicated that in the early second postoperative follow-up (1 month after implantation), the scales had already improved significantly (*P* < 0.001 for PSQI and QOLIE-31, *P* = 0.006 for NDDI-E and GAD-7). We did not find any statistically significant difference between patients with comorbidity and those without on the efficacy of any scales in this study. The efficacy of VNS on the four scales above was related to follow-up time, with a slightly rebound at the last two follow-ups. The NDDI-E as well as the GAD-7 scores were related to better seizure control according to the GEE model. Higher stimulation currents over 1 mA did not improve the efficacy of VNS on the comorbid conditions.

**Conclusions:**

VNS implantation significantly improved sleep quality, mental health, and overall quality of life in adult patients with epilepsy. Such effects could be observed shortly after the implantation and were mostly long-lasting. Further research is needed to validate its long-term effects.

## Background

Since the U.S. Food and Drug Administration (FDA) first approved vagus nerve stimulation (VNS) for the treatment of drug-resistant epilepsy in 1997, this technology has undergone significant advancements, continuously improving in efficacy, safety, and accessibility [1]. As a neuromodulation therapy, VNS exerts its antiepileptic effects by delivering intermittent electrical stimulation to the vagus nerve, influencing the cerebral cortex, limbic system, and thalamic reticular formation, and regulating neuronal excitability [2]. The development of VNS has brought new hope to patients with refractory epilepsy, particularly those who are not candidates for surgical resection or have failed multiple antiseizure medications (ASMs) [3]. Initially, VNS was primarily indicated for focal epilepsy, but recent studies have shown that it also has therapeutic potential in generalized epilepsies and epilepsy syndromes, such as Lennox-Gastaut syndrome (LGS) [4, 5]. Moreover, its approved use has been extended to pediatric patients aged four years and older [1, 6]. The introduction of adaptive VNS (aVNS) has enabled devices to automatically adjust stimulation parameters based on the patient’s seizure activity, further enhancing treatment efficacy while minimizing adverse effects [7, 8].

Beyond seizure control, VNS has garnered increasing attention for its role in managing epilepsy-related comorbidities. Research has suggested that many people with epilepsy (PWE) frequently suffer from mood disorders (e.g., depression, anxiety), cognitive impairment, sleep disturbances, and chronic headaches [9, 10]. These comorbidities not only exacerbate disease burden but could also influence seizure frequency, creating a vicious cycle that significantly reduces quality of life (QoL) [11]. In recent years, VNS has been increasingly recognized for its potential benefits in alleviating epilepsy-related comorbidities [12, 13]. Studies have indicated that VNS can modulate key brain regions such as the lateral orbitofrontal cortex (OFC), anterior cingulate cortex (ACC), amygdala, and hypothalamus, thereby influencing emotion regulation, cognitive processing, and sleep stability [14–16]. Some clinical reports have suggested that VNS therapy can reduce depressive and anxiety symptoms in PWE, potentially enhance cognitive functions (particularly in attention, executive function, and memory), and improve sleep architecture, including shortening sleep latency and increasing slow-wave sleep [17–21]. More importantly, these improvements in comorbidities may occur independently of seizure frequency reduction, suggesting that VNS exerts its effects through additional neuromodulatory mechanisms [17, 22].

However, due to the relatively small sample sizes in existing studies and the lack of standardized and well-defined criteria for comorbidity assessment, direct evidence supporting the effects of VNS on epilepsy-related comorbidities remains limited. Larger-scale, more standardized studies are needed to further validate its therapeutic benefits. This study presents a longitudinal cohort study conducted among adult epilepsy patients in China, systematically investigating the dynamic changes in comorbidities and quality of life following VNS implantation. Through a multicenter collaboration, we enrolled a large-scale patient cohort and employed standardized assessment tools, including NDDI-E (Neurological Disorders Depression Inventory for Epilepsy), GAD-7 (Generalized Anxiexy Disorde-7), QOLIE-31 (Quality of Life in Epilepsy-31), and PSQI (Pittsburgh Sleep Quality Index), to comprehensively evaluate comorbid conditions and quality of life. By providing new evidence for the comprehensive efficacy of VNS in epilepsy treatment, this study aims to offer valuable insights for optimizing future therapeutic strategies.

## Methods

### Inclusion and exclusion criteria

The inclusion criteria were as follows:

1. Patients aged over 18 at time for VNS implantation.
2. Diagnosed with epilepsy according to definition of epilepsy revised by International League Against Epilepsy in 2014.
3. Underwent VNS implantation, from 2021 to 2024.
4. Having complete data for at least two follow-ups after implantation.

The exclusion criteria included patients who were unable to answer required questions during follow-up for any reason, those missing two consecutive follow-ups, and patients who experienced serious adverse events after implantation.

### Outcome measures

Initial clinical evaluations included gender, age at onset, seizure type according to ILAE 2017 classification, baseline seizure frequency, age at implantation and comorbid conditions. For specific scales, NDDI-E, GAD-7, QOLIE-31, and PSQI were also evaluated. The comorbid conditions were evaluated by face-to-face interview and were further confirmed by investigations into medical records. Mental or neurological comorbidities were defined according to ICD-10 codes ( where the diagnosis could be classified into ICD Chapter V and VI, including F00–F99 and G00–G99. See details at https://icd.who.int/browse10/2019/en). The follow-up timepoints were designed as follows: 1 week (first follow-up), 1 month (second), 3 months, 6 months, 9 months, 12 months and 15 months after implantation. Changes in seizure frequency was not the focus of this study and will be described in detail in other publications. We simply used the 50% responder, defined as participants who experienced a reduction of over 50% in seizure compared to baseline, to test the interaction of seizure and comorbidity control. For the settings of VNS stimulation, we selected current as the factor that potentially interacts with comorbidities. In this study, we set current ≥ 1 mA as the high parameter and < 1 mA as the low parameter for VNS stimulation. The major clinical outcome is the change in scores on NDDI-E, GAD-7, QOLIE-31, and PSQI from baseline to the last follow-up. The last follow-up was defined as the latest follow-up the patient had finished, excluding those who only had the first follow-up, since most VNS devices were not turned on at that time. Other clinical outcomes include the change in all comorbidities and mental or neurological comorbidities. Also, to investigate how early the effects of VNS on these scales could be observed we performed a comparison from baseline to the second follow-up.

### Quality control

To ensure the quality of data on NDDI-E, GAD-7, QOLIE-31, and PSQI from different centers, all sites received standardized training prior to data collection. The original data were evaluated by Deng Chen and Lina Zhu for initial quality control. Missing data were further verified by contacting the corresponding epilepsy center. Also, the settings for VNS stimulation were evaluated manually to avoid obvious deviations from the standard protocol in VNS treatment. For ethical considerations, the adjustment of antiseizure medicines (ASMs) was not restricted. Any changes in ASMs were evaluated according to the guidelines of the ILAE.

### Statistical analysis

For statistical analysis, the dichotomous variables were analyzed using the Chi-square test or Fisher’s exact test if the number of any event was less than 4. To illustrate the dynamic change before and after implantation, we used McNemar’s test. For continuous variables, we first performed Kolmogorov–Smirnov test for normal distribution. For data with normal distribution, we performed the Student’s *t*-test or paired *t*-test. For data that did not follow a normal distribution, we performed Mann–Whitney U tests. Multivariable analysis and interaction analysis were performed by using the generalized estimation equation (GEE) method.

## Results

### Recruitment and baseline characteristics

**Fig. 1:**
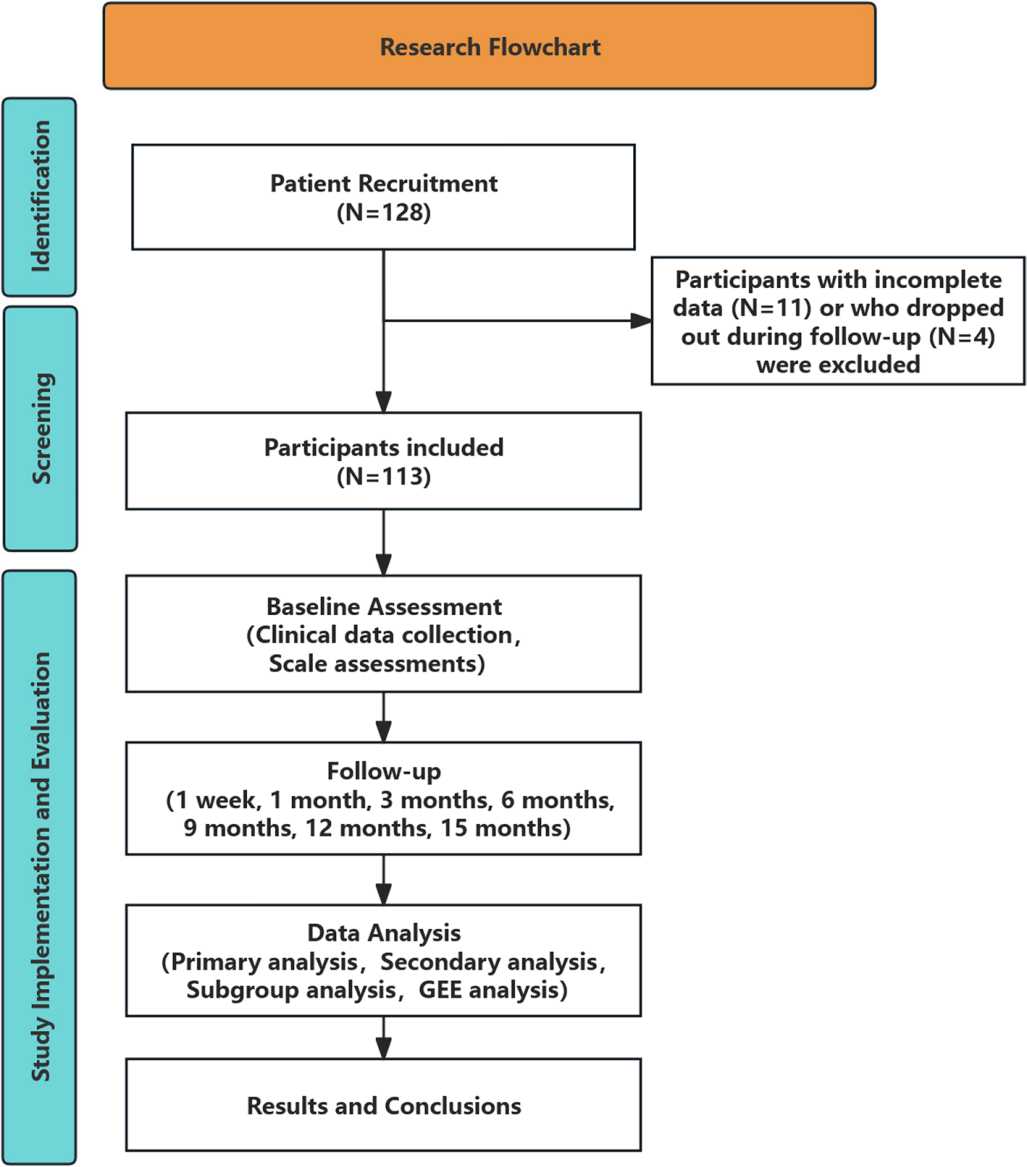
Research flowchart

From 2021 to 2023, a total of 128 participants aged over 18 from 83 hospitals underwent VNS implementation. Among them, 4 dropped out during follow-up after implementation, 11 did not have consecutive complete data and thus were excluded from further analysis. Finally, 113 PWE were enrolled in the analysis. (Fig. 1.) Among them, female patients accounted for 39.82%, while male patients accounted for 60.18%. The average age of onset was 14.31 ± 11.39 years, and the average age at VNS implantation was 28.28 ± 11.25 years. In terms of epilepsy types, focal seizures were the most common (53.98%), followed by generalized seizures (39.82%). Additionally, 53.98% of patients had comorbidities at the time of VNS implantation, with neurological comorbidities being the most prevalent (41.59%). The baseline characteristics are shown in Table 1.

**Table 1:** Baseline characteristics of included participants

	**Total N = 113**			**With Comorbidity N = 61**			**Without comorbidity N = 52**			***P* value**
	*n*/Avg	StdDev	%	*n*/Avg	StdDev	%	*n*/Avg	StdDev	%	
Female	45		39.82%	26		42.62%	19		36.54%	0.51
Age at onset	14.31	11.39		13.20	11.63		15.62	11.08		0.08
Age at implantation	28.28	11.25		27.40	10.95		29.31	11.61		0.26
Number of ASM	2.34	1.24		2.41	1.28		2.25	1.19		0.74
Focal onset	61		53.98%	32		52.46%	29		55.77%	0.73
Generalized onset	45		39.82%	24		39.34%	21		40.38%	0.91
Unknown onset	8		7.08%	6		9.84%	2		3.85%	0.28
With both focal and generalized onset	1		0.88%	1		1.64%	0		0.00%	-
FBTCS	45		39.82%	26		42.62%	19		36.54%	0.51
Baseline seizure frequency	10.90	31.06		12.37	39.03		9.18	17.93		0.87
PWE with comorbidities at implantation	61		53.98%							
Mental comorbidities	26		23.01%							
Neurological comorbidities	47		41.59%							
Other comorbid conditions	2		1.77%							

### Comparison between baseline and the last follow-up

To evaluate the general effect of VNS in real-world data, we compared the last follow-up to baseline. We revealed significant improvements in patients' sleep quality, mental health, and overall quality of life. The PSQI score decreased from 5.43 ± 3.60 to 4.44 ± 3.14 (*P* < 0.001), indicating better sleep quality, while depressive symptoms (NDDI-E) and anxiety symptoms (GAD-7) also showed marked reductions, with scores dropping from 6.49 ± 4.67 to 4.83 ± 4.37 (*P* < 0.001) and from 7.15 ± 5.06 to 4.95 ± 3.69 (*P* < 0.001), respectively. Additionally, the QOLIE-31 score increased from 54.40 ± 15.70 to 61.33 ± 16.19 (*P* < 0.001), suggesting an enhanced quality of life. The VNS stimulation parameters and other details are presented in Table 2.

**Table 2:** Comparison between baseline and the last follow-up

	**Baseline**	**Last follow-up**	***P***	**RD**
PSQI	5.43 ± 3.60	4.44 ± 3.14	< *0.001*	0.46–1.52
NDDI-E	6.49 ± 4.67	4.83 ± 4.37	< *0.001*	1.03–2.28
GAD-7	7.15 ± 5.06	4.95 ± 3.69	< *0.001*	1.41–3.00
QOLIE-31	54.40 ± 15.70	61.33 ± 16.19	< *0.001*	-9.18−-4.67
No. of PWE with comorbidity	61	61		
No. of new PWE with comorbidity		8		
No. of resolve PWE with comorbidity		8	1.00	
Neurological & Phychological (N&P) comorbidity	59	60		
New onset N& P comorbidity		8		
New resolved N& P comorbidity		7	1.00	
50% Respond rate for seizure		46.02%		
Current (mA)		1.49 ± 0.55		
Frequency (Hz)		30 (30, 30)		
Pulse width (ms)		500 (250/500, 500)		
On time (s)		30 (30,30)		
Off time (min)		5 (5,5)		

### Comparison between baseline and the second follow-up

To illustrate whether the VNS could swiftly influence the comorbidities, we compared baseline data to the second postoperative follow-up (after activation, at 1 month after surgery). The result also demonstrated notable improvement in patients'sleep quality, emotional well-being, and overall life satisfaction. The PSQI score showed a reduction from 5.43 ± 3.60 to 4.95 ± 3.36, indicating improved sleep. Similarly, depressive symptoms (NDDI-E: 6.49 ± 4.67 to 5.79 ± 4.25) and anxiety levels (GAD-7: 7.15 ± 5.06 to 6.28 ± 4.26) decreased, while quality of life, as measured by the QOLIE-31 score, rose from 54.40 ± 15.70 to 57.47 ± 15.43. These changes were statistically significant (PSQI and QOLIE-31 with *P* < 0.001, NDDI-E and GAD-7with *P* = 0.006), highlighting the positive effect of VNS in the very early period after implantation. These findings are summarized in Table 3.

**Table 3:** Comparison between baseline and the second follow-up (1 month after implantation)

	**Baseline**	**Second follow-up**	***P***	**RD**
PSQI	5.43 ± 3.60	4.95 ± 3.36	< *0.001*	0.11–0.89
NDDI-E	6.49 ± 4.67	5.79 ± 4.25	*0.006*	0.20–1.20
GAD-7	7.15 ± 5.06	6.28 ± 4.26	*0.006*	0.25–1.48
QOLIE-31	54.40 ± 15.70	57.47 ± 15.43	< *0.001*	-4.70−-1.43
No. of PWE with comorbidity	61	63		
No. of new PWE with comorbidity		7		
No. of resolve PWE with comorbidity		5	1.00	
Neurological & Phychological (N&P) comorbidity	59	61		
New onset N& P comorbidity		6		
New resolved N& P comorbidity		4	1.00	
50% Respond rate for seizure		19.47%		

### Subgroup comparison in patients with and without comorbidity at baseline

We compared patients with and without comorbidities. In the baseline characteristics, no significant differences in gender distribution (*P* = 0.51), age at seizure onset (*P* = 0.08), age at VNS implantation (*P* = 0.26), seizure types (*P* = 0.73), number of ASMs used (*P* = 0.74), or baseline seizure frequency (*P* = 0.87) were found between two groups, suggesting high comparability. For clinical outcome, the changes in PSQI, NDDI-E, GAD-7 and QOLIE-31 from baseline to the last follow up did not differ in two groups. Detailed information is also summarized in Table 1 and Table 4.

**Table 4:** Subgroup comparison between groups with and without comorbidities

	**With comorbidity (N = 61)**	**Without comorbidity (N = 52)**	***P***
ΔPSQI	1.34 ± 2.71	0.58 ± 2.93	0.151
ΔNDDI-E	1.92 ± 3.48	1.35 ± 3.24	0.371
ΔGAD-7	2.30 ± 5.05	2.10 ± 3.13	0.806
ΔQOLIE-31	5.75 ± 13.05	8.29 ± 10.82	0.268

### General estimation equation for testing effect of follow-up time and seizure control on comorbidities

To demonstrate whether the effects of VNS on comorbidities changes according to time (visit number in this study) and whether they were related to seizure control or higher setting of VNS stimulation (which was designed in Method as the current), we applied generalized estimation equation (Table 5). In the tests for interactions, the model for GAD-7 revealed interaction between visit number and 50% responder. Thus, we distinguished the comparison for GAD-7 into two models, where the first model included visit number and current, while the second model included 50% responder and current. No other interactions were found, so for PSQI, NDDI-E or QOLIE-31, the whole model was applied.

**Table 5:** Generalized estimation equation evaluating the effect of visit No., seizure control or stimulation parameter (current) on comorbidities

Interaction *P* Value		Visit No.* 50% responder		50% responder * Higher current ≥ 1 mA		Visit No.* Higher current ≥ 1 mA												
PSQI		0.725		0.074		0.087												
NDDI-E		0.227		0.761		0.104												
GAD-7		*0.018*		0.308		0.447												
QOLIE-31		0.831		0.821		0.943												
Visit No.		Baseline		2		3		4		5		6		7		*P* for the effect of GEE models		
Number of patients		113		113		98		98		81		64		40				
No. of 50% responder		14		22		26		27		29		30		25				
		Score	*P*	Score	*P*	Score	*P*	Score	*P*	Score	P	Score	*P*	Score	*P*	*P* for visit No.	*P* for 50% responder	*P* for higher current
PSQI		5.43 ± 3.60	Ref	4.95 ± 3.36	*0.023*	4.97 ± 3.33	*0.039*	4.60 ± 3.04	*0.003*	4.49 ± 2.97	*0.011*	4.89 ± 3.26	0.122	5.25 ± 3.02	0.588	*0.021*	0.290	0.301
NDDI-E		6.49 ± 4.67	Ref	5.79 ± 4.25	< *0.001*	5.50 ± 4.38	< *0.001*	5.23 ± 4.53	< *0.001*	5.33 ± 4.63	*0.033*	5.17 ± 4.50	*0.020*	5.80 ± 4.15	0.097	*0.002*	*0.049*	0.522
GAD-7	Model 1#	7.15 ± 5.06	Ref	6.28 ± 4.26	*0.016*	6.02 ± 4.13	*0.003*	5.68 ± 4.27	*0.002*	5.52 ± 3.94	< *0.001*	5.41 ± 3.73	*0.018*	5.975 ± 3.72	0.081	*0.010*	N/A	0.060
	Model 2#				N/A		N/A		N/A		N/A		N/A		N/A	N/A	*0.010*	0.070
QOLIE-31		54.40 ±15.70	Ref	57.47 ± 15.43	*0.001*	58.68 ± 15.71	< *0.001*	59.49 ± 15.67	< *0.001*	60.05 ± 16.43	*0.009*	61.03 ± 15.83	*0.006*	59.43 ± 15.78	0.449	< *0.001*	0.210	0.587

Model 1# includes visit No. and VNS parameter (higher current ≥ 1 mA)

Model 2# includes seizure control and VNS parameter (higher current ≥ 1 mA)

In the models, the overall *P* values for visit number were significant for all scales as PSQI (*P* = 0.021), NDDI-E (* P* = 0.002), GAD-7 (* P* = 0.010) and QOLIE-31 (*P* < 0.001), with all the scales improved over time but slight trend of rebounds in the senventh follow-up for PSQI. In terms of 50% responder, its effect on NDDI-E (*P* = 0.049) and GAD-7 (*P* = 0.010) were significant. No statistically significant were detected for current on any scales.

## Discussion

This is a longitudinal real-world study investigating the effects of VNS implantation on comorbid condition and quality of life in adult patients with epilepsy. We also evaluated the efficacy of VNS on comorbidity and its dynamic change over follow-up by GEE model.

We found that after VNS implantation, patients experienced significant improvements in scales on sleep quality, emotional health, anxiety, and depression symptoms, indicating that VNS has a unique therapeutic effect in alleviating epilepsy-related mental comorbidities. More importantly, these effects could be observed in very early follow-up after implantation and were long-lasting to the end of follow-up. Also, there was no interactions between follow-up time and seizure control in GEE model.

For specific comorbidities, the improvement in sleep quality was notable. The study found that after VNS implantation, patients' PSQI scores significantly decreased by 20% at the end of follow-up, indicating a marked improvement in sleep quality. Sleep disorders are common comorbidities in epilepsy patients, who often experience difficulties with falling asleep, maintaining sleep, and disrupted sleep structure. These issues not only affect patients' daily lives but may also worsen the frequency and severity of seizures, creating a vicious cycle [23, 24]. VNS may play a significant role in alleviating sleep disorders in epilepsy patients by modulating neural activity in brain areas such as the cerebral cortex and limbic system [22]. The cerebral cortex is closely related to sleep regulation, while the limbic system involves emotions, memory, and emotional responses. VNS may also improve sleep structure, increase deep sleep (slow-wave sleep) proportion, shorten sleep onset time, and reduce nighttime awakenings, thus enhancing sleep quality [21, 25]. Furthermore, research shows that VNS can help restore the normal levels of neurotransmitters (such as norepinephrine and GABA), further promoting sleep stability and quality [26, 27]. Improved sleep quality also helps control seizures in the long term, which is another advantage of VNS according to our data.

Secondly, the improvement in emotional health by VNS is one of its great values in epilepsy treatment. Existing studies have confirmed the short-term benefits of VNS for emotional health, but our data provid evidence for its long-term effects. Emotional disorders, particularly depression and anxiety, are common comorbidities in epilepsy patients and can severely affect their quality of life and social functioning. The NDDI-E is a tool specifically used to assess depression symptoms in epilepsy patients. The study found that after VNS treatment, patients' NDDI-E scores significantly decreased, indicating relief from depression symptoms. This improvement may be related to the modulation of the limbic system by VNS, particularly its effects on key emotional regulation areas such as the amygdala, hippocampus, and prefrontal cortex [28]. The GAD-7 is used to assess anxiety symptoms. The study showed that after VNS treatment, patients' GAD-7 scores significantly decreased, indicating relief from anxiety symptoms. The improvement in anxiety symptoms may be related to VNS's modulation of the hypothalamic–pituitary–adrenal (HPA) axis, reducing the release of stress hormones (such as cortisol) and thus alleviating anxiety [29, 30]. Additionally, VNS may enhance GABAergic neurotransmission, inhibiting the activity of overexcited neurons, thereby reducing anxiety [31].

The significant improvement in the overall quality of life of patients after VNS treatment is another key finding of this study. The substantial increase in QOLIE-31 scores reflects comprehensive improvements in patients' quality of life, particularly in terms of emotion, cognition, and sleep. The enhancement in quality of life is not only related to the reduction in seizure frequency but also results from multiple factors. Additionally, the overall improvement in quality of life is closely linked to patients' psychological recognition of treatment effects and improved treatment adherence. VNS provides a new treatment option, especially for those whose seizures cannot be controlled by medication. VNS not only helps control seizures but also brings benefits in other aspects. The positive changes experienced by patients during treatment may increase their confidence in treatment and adherence, leading to greater satisfaction and happiness, which further promotes the improvement of quality of life.

In terms of factors related to the efficacy of VNS on comorbid conditions, seizure control (50% responder) for NDDI-E and GAD-7 as well as follow-up times (Visit No.) for all the scales were identified in GEE model. Further interaction analysis revealed that these two factors were mostly independent, except in GAD-7. This stresses three issues. First, participants with better seizure control after VNS surgery could be accompanied by more improvement on depression and anxiety, but the improvement in sleep or life quality seems independent on seizure control. Different studies for VNS have not reached an agreement to the role of seizure control on comorbidities. In Elger G’s findings [17], the relief of depression was independent of seizure control. However, there were only 11 participants in that study, and none of them were tested using NDDI-E or GAD-7. Our GEE model revealed seizure control contributed to NDDI-E and GAD-7 improvement. It is established that better seizure control improves mood in clinical practice [32]. As for sleep quality and quality of life, we did not find a solid correlation with seizure control in our model, and thus the improvement in these two scales was due to VNS alone. Second, when looking at the long-term effect of VNS over time, we observed an overall improvement for all scales during follow-up, with a slight trend of rebound in absolute improvement mostly at follow-up visits 6 and/or 7 (Table 5). It is not clear whether this phenomenon reflects real trends of wearing off on efficacy or is simply since too few participants reached the sixth and seventh follow-up. This requires further prolonged observation. Third, for the parameter of VNS stimulation, we explored the effect of stimulation current. A higher current over 1 mA was not more effective compared to those lower than 1 mA on any of the scales. This is in accordance with the previous study [17].

Also, the effect of VNS on comorbidity must not be exaggerated, since the scales only reflected the relief of certain symptoms, but the treatment of VNS was not sufficient to release the patient from diagnosis of comorbid conditions, as the cure for comorbidity was only seen in 8 patients (Table 2.) and it did not (actually far from) reach statistically significant according to McNemar’s test.

This study has some limitations. Firstly, the follow-up times were not even for each participant, which could affect the results. Secondly, too few non-neurological and non-mental comorbidities were reported (*n* = 2), making it unable to analysis. Thirdly, the data were collected from 83 epilepsy centers, which inevitably brought challenge for quality control on original data, especially for the evaluation on scales. Fourthly, changing ASMs in this cohort was permitted due to ethics concerns, but this could indeed bias the results to some degree. Future research should optimize the evaluation standards and follow-up protocols, perform cross-center quality control and unify the assessment methods on non-neurological and non-mental comorbidities.

## Conclusions

In conclusion, this study provides new evidence for the comprehensive efficacy of VNS in epilepsy treatment, particularly in improving comorbidities and quality of life. The results indicate that VNS significantly improves patients' emotional state, sleep quality, and overall quality of life. These findings provide evidence for optimizing epilepsy treatment strategies and lay the groundwork for further exploring the neural regulatory mechanisms of VNS. Future research should continue to focus on the long-term efficacy of VNS in different types of epilepsy and patient populations to promote its broader application in clinical practice.

## Data Availability

The original data of the current study are available from the corresponding author on reasonable request.

## Abbreviations

ACC: Anterior cingulate cortex
ASMs: Antiseizure medications
FDA: Food and Drug Administration
GAD-7: Generalized Anxiexy Disorde-7
GEE: Generalized estimation equation
LGS: Lennox-Gastaut syndrome
NDDI-E: Neurological Disorders Depression Inventory for Epilepsy
OFC: Orbitofrontal cortex
PSQI: Pittsburgh Sleep Quality Index
PWE: Patients with epilepsy
QoL: Quality of life
QOLIE-31: Quality of Life in Epilepsy-31
VNS: Vagus nerve stimulation
